# Effects of green tea extract epigallocatechin-3-gallate (EGCG) on oral disease-associated microbes: a review

**DOI:** 10.1080/20002297.2022.2131117

**Published:** 2022-10-02

**Authors:** Chen Kong, Huili Zhang, Lingfeng Li, Zhihui Liu

**Affiliations:** Hospital of Stomatology, Jilin University, Changchun, Jilin, China

**Keywords:** Epigallocatechin-3-gallate, green tea, anti-infective properties, dental caries, periodontosis

## Abstract

For thousands of years, caries, periodontitis and mucosal diseases, which are closely related to oral microorganisms, have always affected human health and quality of life. These complex microbiota present in different parts of the mouth can cause chronic infections in the oral cavity under certain conditions, some of which can also lead to acute and systemic diseases. With the mutation of related microorganisms and the continuous emergence of drug-resistant strains, in order to prevent and treat related diseases, in addition to the innovation of diagnosis and treatment technology, the development of new antimicrobial drugs is also important. Catechins are polyphenolic compounds in green tea, some of which are reported to provide health benefits for a variety of diseases. Studies have shown that epigallocatechin-3-gallate (EGCG) is the most abundant and effective active ingredient in green tea catechins, which acts against a variety of gram-positive and negative bacteria, as well as some fungi and viruses. This review aims to summarize the research progress on the activity of EGCG against common oral disease-associated organisms and discuss the mechanisms of these actions, hoping to provide new medication strategies for the prevention and treatment of oral infectious diseases, the future research of EGCG and its translation into clinical practice are also discussed.

## Introduction

Tea is one of the most commonly consumed beverages in the world, second only to water. According to the manufacturing process, especially the drying and fermentation methods, tea can be divided into four major varieties: white tea made from young leaves or buds, green tea made from mature unfermented leaves, oolong tea made from partially fermented leaves, and black tea made from fully fermented leaves [1,2]. In recent years, green tea has become more and more popular due to its health benefits, including anti-inflammatory, antioxidant, anti-cancer, antibacterial and promotion of cardiovascular and oral health. It has been used for daily health care in many countries, and its output accounts for about 20% of the total amount of tea in the world [3]. Because the initial cooking process in green tea production destroyed polyphenol oxidase, the polyphenol content was protected [4]. Catechins in polyphenols are considered to be the source of many biological properties of green tea, which include free catechins such as catechin (C), gallocatechin (GC), epicatechin (EC) and epigallocatechin (EGC), and gallocatechins such as epicatechin gallate (ECG), epigallocatechin gallate (EGCG), catechin gallate (CG) and gallocatechin gallate (GCG) [5,6]. In green tea, EGCG and EGC are the most abundant, accounting for about 59% and 19% of the total catechins, respectively. The former constitutes the most effective antibacterial component in catechins and has now become the subject of most studies [7].

The anti-infective properties of tea have been recorded since ancient times. In China, our ancestors found that tea has anti-miasma, heat-clearing and detoxifying effects. According to the Compendium of Materia Medica, ‘the value of tea is to quench thirst and eliminate plague’ [8]. More than 100 years ago, Mc Naught, a British army surgeon, reported that tea can kill the organisms that cause typhoid and brucellosis (*Salmonella typhi* and *Brucella*) [9]. EGCG is one of the earliest tea polyphenols tested for its effect on bacteria. Its effects on *Staphylococcus aureus*, especially methicillin-resistant *Staphylococcus aureus, Streptococcus* and *Escherichia coli*, have always been the research hotspots for its anti-bacterial properties [10]. In fact, numerous studies have shown that EGCG is active against a variety of pathogenic microorganisms, including many Gram-positive and Gram-negative bacteria, some viruses, fungi, and prions. It is a broad-spectrum anti-infective agent [11,12]. In recent years, with the exploration of the potential application of EGCG in the oral field, this compound has been proved to promote and maintain oral health by preventing the deterioration of periodontitis, reducing enamel and dentin erosion, protecting oral mucosa and it also shows activity against oral cancer cells in vitro and potential to improve halitosis [13–17]. More importantly, EGCG has been shown to be active against most oral disease-associated microbes. In vitro studies have shown that EGCG, a component of green tea, has strong anti-bacterial effects on clinically extracted *Streptococcus mutans, Aggregatibacter actinomycetemcomitans, Porphyromonas gingivalis* and *Prevotella intermedia* [18], suggesting that it can be used as an additive of mouthwash or tooth powder to prevent caries and periodontal disease.

Essentially, oral bacterial diseases are opportunistic infections that are not caused by a single species but by groups of species that live harmlessly in the mouth in very low numbers [19]. When the host has a healthy diet and good oral hygiene, microorganisms, mainly bacteria, tend to be in balance to maintain a healthy oral ecosystem. However, under the influence of specific environmental factors, such as high-sugar diet, poor oral hygiene, alcohol and tobacco consumption, stress, hormone imbalance, diabetes, etc., the balance of local microbiota is disrupted. At the same time, a series of interactions between the microbiota and the host occur, which eventually lead to the occurrence of diseases [20]. Since most oral diseases are polybacterial, it is necessary to develop therapeutic regimens that target the microbiome. As the resistance of bacteria to traditional antibiotics expands, there is a growing interest in some substances with antibacterial properties. In the past, researchers have paid more attention to the beneficial effects of EGCG in anti-inflammatory. However, as a natural and easily obtained plant-derived extract, its potential of application in oral infectious diseases is also worth exploring. Based on the different roles of EGCG depending on microbes, this paper reviews the research progress in recent years on the activity and mechanism of EGCG against oral disease-associated microorganisms, including cariogenic and periodontal related microorganisms, as well as some fungi and viruses that cause oral diseases, hoping to provide a theoretical basis for the development of natural oral drugs.

## Activity of EGCG against common oral disease-associated microbes

### Caries-related microbes

Excessive intake of carbohydrates leads to a matrix-rich environment and the consequent accumulation of acid-producing microorganisms, which trigger ecological changes in the cariogenic microbiota and lead to caries [21]. Although caries involves complex microbiota rather than a single ‘pathogen’, *S. mutans, Lactobacillus* and *Actinomyces viscosus*(now members of the species *Actinomyces naeslundii* and *Actinomyces oris*) have been considered to be highly implicated in the development of dental caries and have been intensively studied [22]. They use fermentable sugars in the environment to generate organic acids, which dissolve inorganic enamel and dentin, resulting in subsequent hydrolysis of collagen and exposure of soft infected dentin [23]. The restorative treatments that are widely used in clinical practice lead to inevitable microleakage and subsequent secondary caries [24]. Therefore, various carrier-mediated drugs with antibacterial properties are gradually being developed and put into use [25]. However, the fluoride induces opportunistic growth of fluoride resistant strains and biosafety problems, the silver compounds cause the black stain of dental caries, and the retention of chlorhexidine on the tooth surface leads to tooth staining and the formation of dental calculi [25–27], all of these also pose challenges for the development of related drugs.

#### Streptococcus mutans

In 1924, Clarke discovered *S. mutans* [28], which is now still considered as one of the main cariogenic bacteria in the oral cavity. As a facultative anaerobe, *S.mutans* can survive anywhere in the mouth [29]. They cause demineralization of tooth hard tissue through high acidity, while they themselvse can grow and reproduce at low pH. They ferment sucrose to produce insoluble extracellular polysaccharides, which enhance their adhesion to tooth surfaces and promote biofilm formation [30].

By sorting out the research results so far, we believe that the activity of EGCG against *S.mutans* is mainly reflected in killing it and inhibiting its virulent factors. As early as 1993, ikigai et al. [31] had found that high concentrations of EGCG would irreversibly damage the bacterial plasma membrane. It was observed by field emission scanning electron microscope that the plasma membrane of *S.mutans* was broken and the cytoplasm leaked after treatment with 0.2 mg/ml EGCG [32]. In 2004, Arakawa et al. [33] put forward the idea that ‘EGCG produces hydrogen peroxide in the lipid layer of bacterial plasma membrane’ and believed that this may be related to its bactericidal effect. Now it has also become one of the two main hypotheses to describe the bactericidal mechanism of catechin. Another hypothesis is the ‘membrane destruction hypothesis’, that is, catechin is embedded in the lipid bilayer, resulting in transverse expansion and membrane rupture [34].

Some intracellular and extracellular enzymes produced by *S.mutans* are its important virulent factors. The membrane-bound F_1_F_0_-atpase system maintains the internal pH by pumping protons out of cells, which is thought to be the main reason for the acid resistance of *S. mutans*. Lactate dehydrogenase(LDH) is responsible for the production of lactic acid, which further enhances the virulence of *S. mutans*. EGCG inhibits the F_1_F_0_-atpase and LDH activities of *S. mutans* at the transcriptional and enzymatic levels (50% inhibitory concentration between 15.6 and 31.25 μg/ml), resulting in decreased acidogenicity and acid resistance [35]. The phosphoenolpyruvate-dependent phosphotransferase system(PEP-PTS), a group of enzymes involved in the transport of sugars into bacteria, consists of enzymes on the cell membrane and in the cytoplasm. The experimental results of Han et al. [36] clearly show that EGCG at 0.5–2 mg/ml can inhibit the activity of PEP-PTS and reduce the uptake of glucose by bacterial cells in a short time, thereby inhibiting the growth of *S. mutans* and reducing the production of acid.

Glucosyltransferase(GTF) uses the glucose part of sucrose as substrate to synthesize glucan, which makes it possible for bacteria to adhere to enamel and microorganisms to adhere to each other [37]. *S. mutans* produces three GTF: GTF-B, -C, and -D. These polymers, especially the α-1, 3-glycosidically linked water-insoluble glucans, are major constituents of plaque biofilm matrix [38]. Previous studies have shown that 250 μg/ml EGCG could significantly reduce the biomass and acid production of *S. mutans* biofilm [39]. Xu et al. [40] found that EGCG concentrations in the range of 7.8–31.25 μg/ml exhibited a dose-dependent inhibition of the initial attachment of *S. mutans*, and EGCG at sub-minimum inhibitory concentration(MIC) level(15.6 μg/ml) significantly inhibited the expression of gtf-B, C, D genes. Similar results were obtained by Schneider-Rayman et al. [41]. They also demonstrated that EGCG reduced the expression of nox and sodA genes involved in oxidative stress protection, and could immediately cause changes in membrane potential. This study revealed that EGCG has both antibacterial activity against *S. mutans*, such as changes in membrane potential and EGCG-induced protein precipitation, leading to the loss of their biological activity, and anti-biofilm activity, such as direct inhibition of genes involved in biofilm formation. These two activities are mediated by different mechanisms. Although reduced bacterial growth may lead to reduced biofilm formation, EGCG directly affected the expression of genes that regulate biofilm formation, and the minimum biofilm inhibitory concentration(MBIC) of EGCG was significantly lower than its MIC. Another study also showed that EGCG inhibited the formation of *S. mutans* biofilm and destroyed the formed biofilm, which was not mediated by interaction with *Streptococcus* lipoteichoic acid (LTA) [32].

In a word, EGCG destroys bacterial cell membrane to kill bacteria. At the same time, it inhibits a variety of intracellular and extracellular enzymes produced by *S. mutans*, including F_1_F_0_-atpase, LDH, PEP-PTS, GTF, etc., to reduce its acidogenicity and acid resistance, inhibit its growth, adhesion, aggregation and other physiological activities, and interfere with the formation of plaque biofilm. Despite extensive in vivo validation, it remains unclear whether EGCG can exert beneficial activities in the complex oral environment. A study showed that the reduction rate of *S. mutans* in the saliva of children before and after gargling with EGCG solution was 79.9% [42]. But this is only a short-term effect. Considering that the maintenance of oral health does not require the complete elimination of a specific bacteria, but rather the restoration of the proportion of resident microbiota, more in vivo experiments are warranted to assess the long-term effects of EGCG on the oral micro-ecosystem.

#### Lactobacillus

*Lactobacillus* is a member of the normal oral flora and can be isolated from the oral cavity of healthy individuals, accounting for about 1% of the oral culturable microbiota [43]. It has strong acid resistance, can continue to survive in a strong acid environment and ferment sugar to produce acid, resulting in demineralization of tooth hard tissue [44]. Because they cannot form plaque on the tooth surface by themselves, they need to rely on the extracellular polysaccharides produced by other oral organisms (mainly *Streptococcus*) for colonization. In recent years, scholars at home and abroad generally believe that they actively participate in the development of caries rather than the initial process [45,46]. It is reported that some species of *lactobacilli* are abundant in caries sites, especially in deep dentin caries [47]. It is now commonly used as a microbial marker to assess the risk of dental caries [48].

Numerous studies have shown that gargling with green tea extract every day can reduce the amount of *Lactobacillus* in saliva [49–51]. The MIC and minimum bactericidal concentration(MBC) of green tea extract against *Lactobacillus acidophilus* in vitro were 0.3% and 0.9%, respectively [52]. An in vivo study demonstrated that the reduction percentage of *Lactobacillus* caused by gargling with EGCG solution alone (72.09%) was higher than that of green tea (59.17%), but both were lower than that of chlorhexidine (86.02%) [42]. Tea polyphenols have been shown to effectively inhibit the growth and acid production of *Lactobacillus* [53]. In order to explore the effect of tea polyphenols on the initial adhesion of major cariogenic bacteria to type I collagen, Xiao et al. [54] prepared an in vitro model of experimental film(C-HA) using hydroxyapatite and type I collagen. The results showed that no matter C-HA or bacteria were pretreated with tea polyphenols, 1.0–4.0 mg/ml tea polyphenols solution could effectively inhibit the adhesion of *Lactobacillus* to C-HA, and the inhibition rate was dose-dependent. This indicates that tea polyphenols may interfere with the combination of bacteria and collagen by changing the properties of collagen surface and interacting with bacterial surface adhesins to reduce the adhesion of bacteria to C-HA. However, in the above experiments, it is impossible to determine whether the reduction of *Lactobacillus* is due to the inhibitory effect of EGCG on *Streptococcus* that aids its colonization, and there is also a lack of research on each single component of tea polyphenols. Therefore, it cannot be directly proved that EGCG has antibacterial activity against *Lactobacillus*.

Although *Lactobacilli* are considered to have cariogenic potential, their positive effects in promoting the balance of oral flora and maintaining oral health have also been confirmed. It is well known that *Lactobacillus* is considered a probiotic [55], and some oral Lactobacilli are able to inhibit the growth of caries and periodontitis-related microbes such as *S. mutans, P. gingivalis*, and *P. intermedia* in vitro [56,57]. A randomized, double-blind, placebo-controlled intervention study demonstrated that long-term consumption of milk containing *Lactobacillus rhamnosus* reduced the incidence of early caries in kindergarten children [58]. In the study of Zhang et al. [59], both tea extracts EGCG and GCG exhibited growth-promoting effects on this probiotic. Later studies also proved that EGCG can enhance the viability of *L. plantarum, L. fermentum, L. acidophilus* and *L. gasseri* and stimulate their growth [60,61]. These results may be related to the sensitivity of bacteria to EGCG. Higuchi et al. [62] found that 2.5 mg/ml EGCG inhibited the growth of *P. gingivalis, P. intermedia* and *Fusobacterium nucleatum*, 5 mg/ml EGCG inhibited the growth of *S. mutans*, 10 mg/ml EGCG inhibited the growth of *A. actinomycetemcomitans*, and 25 mg/ml EGCG inhibited the growth of *L. salivarius WB21*. This suggests that the pros and cons of EGCG on oral health may depend on the ingested dose. Low dose may inhibit oral pathogenic bacteria and promote the growth of beneficial microbiota. If the concentration of EGCG that inhibits disease-associated microorganisms without inhibiting *Lactobacillus* can be determined, the combination of EGCG and *Lactobacillus* may have a more potent and extensive positive effect on the oral environment.

In summary, the current research can not clearly judge the effect of EGCG on *Lactobacillus*, and its influence mechanism needs exploring urgently. Our understanding of the role of *Lactobacillus* in oral microbiota is also far from sufficient. *Lactobacillus* seems to exhibit high resistance to EGCG in vitro, but different doses of EGCG in vitro studies directly affect the underlying molecular mechanisms, leading to different antimicrobial effects. And extreme doses also stress other cells in some way. Inappropriate doses introduced into the body can cause another microbiota imbalance. Overall, the evaluation of appropriate concentrations, the long-term tracking of in vivo effects, and the potential value of EGCG combined with probiotics are all worthy of further exploration.

#### Actinomyces

Common *Actinomyces* in the oral cavity include *Actinomyces israelii, Actinomyces naeslundii, Actinomyces oris* and *Actinomyces odontolyticus. A. oris* and *A. naeslundii* can cause root caries in animal experiments [63]. There are two types of fimbriae in *A. oris*. Type I fimbriae are mainly involved in the adhesion of bacteria to the tooth surface,and type II fimbriae mainly mediates the aggregation of bacteria and plaque formation [64]. *Actinomyces* can colonize a range of matrix surfaces in the oral cavity, including enamel and other oral mucosa, and play an important role in a series of oral diseases, including caries, periapical disease, periodontitis, actinomycosis, alveolar bone lesions and so on [65]. At present, there is no final conclusion about the role of *Actinomyces* in the occurrence and development of dental caries.

Green tea extract has been shown to have antibacterial activity against *A. oris* with a MIC of 0.06 mg/mL [66]. Tea polyphenols with a concentration lower than or equal to 8 mg/ml can effectively inhibit the growth of *A. oris* and its acid production [67]. Wang et al. [68] found that EGCG inhibited the attachment of *A. naeslundii* to hard surfaces such as glass and stainless steel by reducing the hydrophobicity of the cell surface. It is speculated that the hydrogen bond formed between the cell and the matrix surface was disrupted due to EGCG binding to cell surface ligands, but this did not occur on the hydroxyapatite surface, probably because EGCG has an affinity for hydroxyapatite. The results of an in vitro study showed that there was no statistical difference in the attachment of *A. naeslundii* to human gingival fibroblasts before and after EGCG treatment [69].

In conclusion, the effects of green tea extract or tea polyphenols rich in multiple chemical constituents on *Actinomyces* seem to be more significant than the use of single EGCG. However, more studies are needed to further identify the specific active ingredients and their mechanisms of action.

### Periodontal disease-related microbes

Periodontal status is thought to be closely related to changes in gingival sulcus microbiota. Periodontal tissue is continuously exposed to oral microbiota and physicochemical stimuli from chewing and breathing, and a delicate balance is maintained between local immune responses and the microbiota under physiological conditions. Under the influence of environmental factors and host susceptibility, some specific microorganisms increase, causing dysbacteriosis, the increase of pathogenicity of the whole community, and the excessive activation of the host immune response, which eventually leads to the destruction of periodontal tissue [70,71]. It is generally believed that if the clinical symptoms of periodontal disease persist after thorough mechanical treatment, such as attachment loss and exploratory bleeding,a combination of drug therapy should be considered to control plaque and inflammation [72]. However, the frequent recolonization of periodontal pathogens to the treatment site and the emergence of antibiotic resistance have led to the exploration of new drugs and methods for periodontal disease treatment in recent years [73].

#### Porphyromonas gingivalis

*P. gingivalis*, a gram-negative anaerobic bacterium, is considered as the ‘key microorganism’ of chronic periodontitis [74,75]. They accumulate locally and express a series of virulence factors, including lipopolysaccharide, collagenase, gingival protease and fimbriae, which cause tissue damage directly or indirectly, and escape host immune surveillance by invading cells and tissues [76]. Heme is the only source of iron and protoporphyrin IX of *P. gingivalis*, which is essential for their growth and survival in periodontal pockets [77].

An in vivo study shows that continuous oral administration of EGCG alleviates periodontitis in mice caused by *P. gingivalis* [78]. But this study focused on proving the anti-inflammatory activity of EGCG, and no suitable animal model for evaluating the antibacterial effect has been found. In vitro, EGCG was observed to destroy the cell membrane and cell wall of *P. gingivalis*, inhibit the formation of biofilm and destroy the established biofilm [79]. The MIC and MBC of EGCG against *P. gingivalis* are 97.5 µg/mL and 187.5 µg/mL, respectively. At sub-MIC level, it can reduce CH_3_SH production by inhibiting *mgl* mRNA and protein expression [80]. *mgl*, the gene encoding L-methionine-α-deamino-γ-mercaptomethane lyase, is responsible for the production of methylthiol (CH_3_SH) by oral anaerobic bacteria, which proves the potential of EGCG in reducing halitosis caused by volatile sulfur compounds (VSC).

Gingival proteases are a group of cysteine proteases on the cell surface of *P. gingivalis*. They are important virulence factors, accounting for 85% of the total proteolytic activity of *P. gingivalis*. They can degrade a variety of host proteins, including integrin-fibronectin binding, cytokines, immunoglobulins and complement factors, etc [81]. Depending on different substrates, they can be divided into arginine-specific(Arg-X) and lysine-specific(Lys-X) gingival proteases. Arg-X includes RgpA containing one proteolytic domain and one adhesion domain and RgpB containing only one proteolytic domain. There is only one type of Lys-X, the Kgp that contains one proteolytic domain and one adhesion domain [82,83]. In the study of Okamoto et al. [84], EGCG significantly inhibited the Rgp activity of *P. gingivalis*, and also showed a lesser degree of inhibition on Kgp activity. This inhibitory activity was only observed in catechins containing the galloyl moiety.

Sakanaka et al. [85,86] found that EGCG at a concentration of 250–500 μg/ml could completely inhibit the growth and adhesion of *P. gingivalis* on oral epithelial cells. And it could inhibit the production of its toxic end metabolites, which are known to easily penetrate into the periodontal tissue from the periodontal pocket, disrupt the cellular activity and defense system of the host. The results showed that in general anaerobic medium, 0. 5 mg/mL EGCG inhibited the production of phenylacetic acid. The resting cells of *P. gingivalis* lack the ability to grow, but they can still produce phenylacetic acid by metabolizing various substrates. This ability was also completely inhibited by EGCG. Since the above-mentioned activities were also only expressed in catechins containing galloyl moiety, it is speculated that some inhibitory effects of EGCG may be related to the existence of galloyl groups, which are linked to the 3-OH of the catechin or epicatechin moieties.

Previous experiments have demonstrated that both aqueous and 50% ethanolic extracts of catechins strongly inhibited the collagenolytic activity of *P. gingivalis* and reduced cytotoxicity to human gingival fibroblasts [87], but did not explore specific active components or groups. Later, Makimura et al. [88] added various green tea catechins to the reaction mixture containing collagenase and collagen, respectively. They found that ECG and EGCG had the strongest inhibitory effect on collagenase activity, and gallyl-containing catechins could completely inhibit collagenase activity in gingival crevicular fluid from aggressive periodontitis in adults, while C, EC, EGC and GC did not show any collagenase inhibition, suggesting the role of galloyl structure in the inhibition of collagenase from eukaryotic and prokaryotic cells.

Fimbriae is another powerful virulence factor of *P. gingivalis*, which can promote the adhesion of bacteria to salivary proteins, extracellular matrix, eukaryotic cells and the same or other kinds of bacteria, and mediate the formation of biofilm. Type I(major) fimbriae plays an important role in colonization and invasion,the *fimA* gene encodes its major subunit, and type II(minor) fimbriae have a higher pro-inflammatory capacity [89,90]. Meanwhile,*P. gingivalis* can actively invade gingival epithelial cells and maintain viability and replication in them [91]. This invasive ability also derives from its type I fimbriae, which bind to β1 integrin on the host cell surface to promote adhesion and lead to remodeling of the actin and tubulin cytoskeleton to allow internalization [92,93]. Fournier-Larente et al [94] found that EGCG dose-dependently inhibited the adhesion of *P. gingivalis* to oral epithelial cells. On the one hand, EGCG inhibits the expression of some genes involved in host colonization(*fimA, hagA, hagB*), tissue destruction(*rgpA, kgp*) and heme acquisition(*hem*). Reducing the expression of *fimA* may also help to reduce inflammation, because this virulence factor has the ability to induce host cells to produce cytokines. On the other hand, EGCG increased the expression of the stress protein *htrA* gene. The periplasmic high temperature requirement A protein(HtrA) is known to be responsible for resisting oxidative stress in *P. gingivalis*, helping them survive under stressful conditions [95], which indicates that EGCG exerts a stress on the bacteria.

To sum up,EGCG not only damages the cell membrane and cell wall of *P. gingivalis*, but more importantly, it affects the growth and adhesion of *P. gingivalis*, interferes with its biofilm formation, weakens its invasiveness to host cells and tissues, and reduces the production of VSC and the resulting halitosis by inhibition of relevant virulence factors (gingival protease, collagenase, toxic end metabolites, fimbriae, etc.). Most of the current researches related to periodontal pathogens are based on one pathogen or single species biofilm. Exploring the interaction between two or more bacteria and studying the efficacy of EGCG in more complex microbial ecosystems should become the direction of future research.

#### Aggregatibacter actinomycetemcomitans

*A. actinomycetemcomitans* is a gram-negative bacterium closely associated with the development of locally aggressive periodontitis. Leukotoxin (Ltx) is an important virulence factor secreted by them. It exists in two forms, one is free soluble protein, and the other is membrane-soluble Ltx related to outer membrane vesicles(OMVs) [96]. The former binds [97]** to cholesterol on host cells and the β2 integrin receptor lymphocyte function-associated antigen 1(LFA-1), triggering internalization and subsequent cell death, the latter is transported to host cells by cholesterol-and receptor-independent mechanisms. Both disrupt the host immune response, making it easier for bacteria to colonize in tissues[[98-]]. Inhibition of Ltx activity is considered to be the key to reducing the pathogenicity of *A. actinomycetemcomitans*.

EGCG has definite antibacterial activity against *A. actinomycetemcomitans* [99,100], but its effect on Ltx secretion and activity is complex. When EGCG and Ltx were co-cultured with HL60 cells, EGCG significantly inhibited the lysis of HL60, indicating that EGCG can reduce the cytotoxicity of free Ltx [101]. Later, Saito et al. [102] co-cultured EGCG and Ltx-containing vesicles with human monocyte THP-1 cells, they found that EGCG also inhibited the lysis of THP-1 cells, indicating that EGCG also inhibited vesicle-related Ltx. They labelled *A. actinomycetemcomitans* vesicles with fluorescent dyes and found that when EGCG, vesicles, THP-1 cells were co-cultured, or vesicles pretreated with EGCG were co-cultured with THP-1 cells, the lysis of THP-1 cells was significantly reduced, and their cytoplasmic membranes were rarely fluorescently labeled. However, when THP-1 cells pretreated with EGCG were co-cultured with vesicles, the lysis was not inhibited. Therefore, that EGCG inhibits the interaction between vesicles and THP-1 cells or the secretion of Ltx in vesicles by binding with vesicles is considered to be the possible mechanism of its action.

Chang et al. [103] deeply studied the inhibitory mechanism of catechin on free Ltx. Since compounds that reduce membrane fluidity can prevent toxins from entering the cell membrane to reduce toxin activity [104], and all three gallylated catechins (EGCG, ECG, and GCG) can significantly reduce the membrane fluidity of THP-1 cells, they speculated that if this is why catechin inhibits the activity of Ltx, pretreatment of THP-1 cells with catechin before adding Ltx should show a stronger inhibitory effect. In this case, however, they only observed a similar inhibitory effect as adding catechin and Ltx to the cells simultaneously. When they pretreated Ltx with various catechins separately, and then added the mixture to THP-1 cells, the inhibitory effect was greatly enhanced. Three gallylated catechins exhibited the greatest inhibitory effect on Ltx, almost completely blocking its activity. This indicates that catechins act on Ltx rather than cell membrane targets to achieve protective effects. And compared with other catechins, EGCG still retained its inhibitory activity on Ltx when the concentration was reduced by 10-fold and 100-fold. They also found that catechins, especially gallylated catechins, could alter the secondary structure of Ltx proteins, thereby reducing the affinity of Ltx for cholesterol on the host cell membrane, and this interaction is an important initial step in its toxicity.

It is reported that the MIC of EGCG against *A. actinomycetemcomitans* is 10 μg/mL. At sub-inhibitory concentrations(5 μg/mL) that do not affect bacterial growth, EGCG promotes Ltx production, with total Ltx production almost twice as high as in the untreated group, but the amount of Ltx released into the supernatant is lower than that in the untreated group. This is due to the enhanced affinity of Ltx to bacterial cell surface caused by EGCG. Immediately after Ltx is secreted by *A. actinomycetemcomitans* via the type1 secretion system, EGCG promotes its reassociation with the bacterial membrane, which inhibits the release of Ltx from bacterial cells during early growth stages [105]. The amount of Ltx in OMV produced by *A. actinomycetemcomitans* treated with EGCG is about 6 times higher than that in the group without EGCG, which may also be due to the increased binding of Ltx to the bacterial cell surface by EGCG. Another interesting finding is that the initial addition of 5 μg/mL EGCG to the co-culture did not inhibit the toxicity of *A. actinomycetemcomitans* to THP-1 cells, but addition of 5 μg/mL EGCG to the co-culture at 0, 6 and 13 hours each resulted in a significant decrease in cytotoxicity [106]. One possible explanation is that Ltx is continuously produced in the whole co-culture experiment. With the passage of time, the ratio of EGCG/Ltx decreases to an extent that is insufficient to inhibit Ltx activity. Repeated administration of EGCG appears to be more effective in promoting antibacterial effect.

In general, high concentrations of EGCG directly inhibited the growth of *A. actinomycetemcomitans*, while low concentrations of EGCG changed the structure of Ltx, resulting in decreased affinity for cholesterol on the host cell membrane and increased affinity for bacterial cell surface components, thus reducing the toxicity to immune cells in the supernatant. A single low-dose administration of EGCG may not protect host cells from bacterial cytotoxicity, and multiple administration strategies have significantly improved effects. Further refinement may determine the optimal concentration and mode of administration. Since enough in vitro studies have demonstrated the efficacy of EGCG at the molecular and cellular levels, it is necessary to develop animal models and conduct clinical trials in order to more rigorously evaluate its role in mixed infections and obtain more precise and effective information.

#### Other periodontal disease-related microbes

*P. intermedia* has strong enzymatic activity, which is believed to be involved in the development of various periodontal diseases together with *P. gingivalis* [107]. Green tea catechins have obvious inhibitory effect on *P. intermedia*, the MIC is 1 mg/ml, and it has bactericidal effect at high concentration [108]. The researchers found that EGCG can effectively inhibit the protein tyrosine phosphatase activity of *P. intermedia*, and this effect is derived from the galloyl moiety in its structure [109].

*A. israelii* was found to be significantly increased in the gingival sulcus of patients with gingivitis. EGCG also has bactericidal activity against it and can inhibit its biofilm formation to a certain extent [110].

*Fusobacterium nucleatum* is a gram-negative anaerobic bacterium that often causes multibacterial co-infection with other anaerobic or facultative anaerobic bacteria, such as necrotizing ulcerative gingivitis and root canal infection [111]. EGCG has obvious antibacterial activity against planktonic *F. nucleatum* [112]. It is reported that the MIC and MBC of EGCG against *F. nucleatum* are 500 μg/mL and 1000 μg/mL, respectively, and the antibacterial mechanism may involve disruption of bacterial cell membranes and dose-dependent chelation of iron, which is an essential nutrient for bacteria. At the concentration lower than MIC, EGCG did not interfere with the growth of *F. nucleatum* but prevented its biofilm formation, and the addition of 62.5 μg/ml EGCG reduced biofilm formation by 55.4%. EGCG at MBC level could decrease the activity of established biofilms over time. Furthermore, in an in vitro basement membrane model, EGCG reduced the adhesion of *F. nucleatum* to oral epithelial cells and extracellular matrix proteins, and attenuated the hemolytic activity and H_2_S-producing ability of *F. nucleatum. F. nucleatum* provides iron for itself and other microorganisms associated with periodontal disease by lysing red blood cells and releasing hemoglobin, promoting their proliferation in the periodontal pocket, and participates in the occurrence of halitosis through the ability to produce volatile sulfur compounds such as H_2_S, which also have strong toxicity to immune cells and mucosal cells [113].

### Common oral pathogenic fungi

*Candida*, especially *Candida albicans*, is currently recognized as the most clinically significant oral opportunistic pathogenic fungi. Changes in host immunity, stress, resident microbiota and other factors can lead to the overgrowth of *C. albicans*, resulting in a spectrum of *Candida* infections ranging from superficial mucosa to hematogenous dissemination. The formation of biofilm provides an important guarantee for the survival of *Candida* in various adverse conditions [114]. The main problem of antifungals currently used is not their antifungal activity, but the emergence of drug-resistant strains and the potential side effects, since most of them are nephrotoxic or hepatotoxic [115]. Therefore, it is necessary to develop and test less toxic compounds from nature.

EGCG can inhibit a variety of pathogenic *Candida* clinically isolated. Its antibacterial activity against different *Candida* is not the same, but overall slightly higher than the tested antifungal drugs, including amphotericin B, itraconazole, fluconazole, flucytosine and miconazole, etc [116,117]. The antifungal activity of EGCG is pH-dependent. For the tested *C. albicans* strains, the MIC_90_ of EGCG is 2000 mg/L at pH6.0, 500 ~ 1000 mg/L at pH6.5, and 15.6 ~ 250 mg/L at pH7.0 [118].

A study by Navarro-Martínez et al. [119] explored the mechanism of action of EGCG on *C. albicans*. They found that EGCG potently inhibited the activity of dihydrofolate reductase of *C. albicans*. This inhibition was highly regulated by pH and more active at slightly alkaline pH. When combined with azole antifungals(ketoconazole and itraconazole) or ergosterol biosynthetic pathway inhibitors, they exhibit synergistic effects. At the same time, this study provides a possible explanation for the molecular mechanism of EGCG’s antifungal effect: EGCG indirectly interferes with the ergosterol biosynthetic pathway by disrupting the folate cycle, and inhibits ergosterol biosynthesis by inhibiting sterol C-24 methyltransferase through the reduction of S-adenosyl-methionine cell pool. Through electron microscope observation, some researchers found that the cell structure of *C. albicans* treated with EGCG was deformed, the cell wall was broken, and the cell contents were released. And in the molecular docking experiment, EGCG was observed to have a strong interaction with ergosterol, a fungal cell membrane molecule [120].

An in vitro study showed that EGCG, EGC and ECG all inhibited the growth of *C. albicans* biofilm and disrupted the formed biofilm, and EGCG was more active than the other two catechins. Proteasome exists in all eukaryotic cells. In *C. albicans*, it is responsible for regulating metabolism and responding appropriately to environmental signals. The *C. albicans* proteasome regulates 3 major proteolytic activities: trypsin-like, chymotrypsin-like, and peptidyl-glutamyl peptide-hydrolyzing activities. EGCG inhibits the latter two activities, which is a factor leading to the decline of *C. albicans* growth rate and the obstruction of biofilm formation and maintenance. The control group not treated with EGCG could not transport the fluorescent peptide substrate into the cytoplasm, but the cells treated with EGCG for 24 hours could, suggesting that other targets of EGCG may involve the cell membrane or cell wall, or both [121]. Han et al. [122] studied the synergistic effect and mechanism of EGCG combined with amphotericin B in the mouse model of disseminated candidiasis caused by *C. albicans*. BALB/c mice given EGCG intraperitoneally before intravenous inoculation with *C. albicans* yeast cells had a longer mean survival time (MST) than mice given diluent. EGCG treatment inhibited the mycelial formation of *C. albicans* in yeast form and the growth of *Candida* cells. Compared with 0.5 mg/kg amphotericin B alone(MST:11.7d) or 2 mg/kg EGCG alone(MST:13.9d), the survival time of mice given both drugs(MST:42.1d) was significantly prolonged.

In summary, EGCG has obvious antibacterial activity against *Candida*, which may even be higher than that of currently used antifungal agents. EGCG has been reported to enhance the antifungal effects of ketoconazole, miconazole, fluconazole, and amphotericin B, including planktonic and biofilm cells. Combined with fluconazole, EGCG can induce apoptosis of fluconazole-resistant *Candida tropicalis* [120,123,124]. Combined treatment with EGCG may reduce the dose of commonly used antifungal agents, preventing adverse reactions and the emergence of drug-resistant strains. Further research is required at present.

### Common oral viruses

Worldwide, more than 630 million people are infected with human papillomavirus(HPV), which can cause papillomas or warts and malignant tumors in the mucosa, genitals and anus. The prevalence of oral HPV infection differs significantly by geographic location, which is approximately 7% among U.S. adults aged 18 to 69 [125].

Condyloma acuminatum is one of the most common sexually transmitted diseases. It is caused by low-risk HPV, mainly HPV 6 and 11. EGCG shows a strong anti-HPV11 effect and can inhibit the expression of HPV11 *E6* and *E7* mRNA in recombinant HPV11. HaCaT cells. *E6* and *E7* genes play an important role in HPV replication and interaction with host cells leading to disease [126]. Interferon(IFN) is a component of the innate immune system, which prevents viral infection through antiviral, antiproliferative and immune stimulation mechanisms. Type I IFN signal induces the transcription of interferon stimulated gene(*ISG*), whose protein products inhibit the viral life cycle. HPV-2 *E7* inhibited the expression of *ISG* by downregulating the type I IFN signaling pathway. EGCG pretreatment resulted in a dose-dependent decrease in HPV-2 *E7* mRNA expression, and *E7* expression was blocked by inhibiting *E7* transfection, thereby maintaining the expression of *ISG* and components in type I IFN signaling pathway [127]. Similar results were obtained in the experiments of Yap et al. [128], that is, EGCG downregulates the expression of HPV-18 E6 and E7 oncoproteins, thus allowing the re-expression of its target gene *TSG*, leading to the growth inhibition and apoptosis of keratinocytes. But their analysis showed that EGCG treatment did not affect the mRNA levels of *E6* and *E7*, but instead stimulated their protein conversion by enhancing the degradation of E6 and E7 through the ubiquitin-proteasome pathway. In addition to the inhibitory effect on HPV oncogenes and oncoproteins, several studies have shown that EGCG has a series of activities against cancer cells, including anti-proliferation, anti-metastasis and pro-apoptosis. The combination application with other chemical drugs also showed considerable Prospects. Since the focus of this review is to summarize the activity of EGCG on oral pathogens, we would like to recommend the article by Wang et al. [129], which provides a detailed review of the relevant contents.

Herpes simplex virus(HSV) is responsible for some oral and genital herpes, blindness, and encephalitis, which often causes repeated infection. When drug-resistant HSV mutants emerge, use of current antiviral therapies may be limited [130]. It has been reported that 1–2 μM EGCG has virucidal activity against HSV virions at 25–37°C, and 0.5–20 μM EGCG can reduce the plaque formation ability of HSV virions [131]. EGCG inactivates multiple clinical isolates of HSV-1 and HSV-2, and this anti-HSV activity directly targets virions, not cells. On the one hand, EGCG directly inactivates HSV virions by binding to the envelope glycoproteins gB, gD or another envelope glycoprotein [132]. On the other hand, EGCG inhibits the primary attachment of HSV-1 to the cytoglycan heparan sulfate, a key step in viral entry, by interacting with virion surface proteins [133]. Studies have shown that treatment of oral epithelial cells with EGCG at 25 μg/ml, either before or after HSV-1 inoculation, can avoid HSV-1-induced cell death and significantly reduce the amount of virus released. The expression of viral protein infected cell protein 0(ICP0) and ICP5 was greatly inhibited. At the same time, EGCG may synergize with acyclovir(ACV) to reduce the cytotoxic effect of HSV-1. When combined with ACV, the expression of virus ICP5, thymidine kinase and gD decreased significantly. In addition, in the presence of EGCG and ACV, viral DNA replication in infected cells was greatly reduced [134]. A study exploring structure-function relationships through self-organizing graphs and backpropagation neural networks provides new insights into structural effects in the anti-HSV-1 activity of gallylated polyphenols, arguing that electrostatic effects and distances between atoms are closely related to the anti-HSV-1 activity of these compounds [135].

In general, EGCG inhibits cell growth and viral proliferation by inhibiting the expression of HPV oncogenes and oncoproteins, exerts anti-HSV effects by directly acting on virions, and also inhibits the expression of HSV protein and DNA replication. EGCG can be regarded as a potential drug for HPV or HSV infected patients. Further work is required to assess the precise mechanism of EGCG virucidal activity and its application in the treatment of viral infectious diseases.

## Summary and prospect

Caries and periodontal disease are the two most prevalent oral diseases associated with endogenous bacteria. Specific microorganisms are defined as key ‘pathogens’ whose accumulation can alter the local environment and the proportion of other microorganisms in the ecological niche. Most treatments now aim to control their amounts or suppress their virulence. Growing evidences suggest that EGCG has strong and broad-spectrum antibacterial properties. For the flora related to dental caries and periodontal disease, high doses of EGCG mainly kill bacteria by destroying bacterial structures, while low doses exert antibacterial effects by inhibiting important virulence factors, which often leads to bacterial biofilm disruption and growth inhibition, the obstruction of bacterial adhesion and aggregation, the weakening of the ability to absorb nutrients, and the reduction of invasion to tissues. EGCG also showed significant antibacterial activity against oral *Candida*. When EGCG is combined with other antifungal drugs, the antibacterial effect can be enhanced, and adverse reactions and the emergence of drug-resistant strains can be prevented at the same time. For some common oral viruses such as HPV and HSV, the expression of their pathogenic genes and viral proteins are inhibited by EGCG, and HSV is directly inactivated under the action of EGCG. These results demonstrate the potential of EGCG in the treatment of oral infectious diseases. EGCG may be used alone or in combination with other anti-infective drugs to fight against oral pathogens.

Stability, biosafety and availability are the main issues in clinical translation of EGCG. EGCG is not stable under alkaline and neutral conditions. Meanwhile, high water solubility and poor transmembrane hydrophobicity result in low cellular uptake rates. Metabolic transformations such as methylation, glucuronidation and sulfation and active efflux through multidrug resistance-related protein 2 also contribute to the lower bioavailability of EGCG [136]. One of the most common problems in the chemotherapy of caries and periodontal disease is the inability to maintain drug concentrations. When the antibacterial agent is introduced into the oral cavity at the initial concentration, the drug concentration decreases immediately, eventually falling below the MIC. Therefore, some new methods are needed for the sustained delivery of antimicrobial drugs to the local area. In addition, liver injury associated with green tea and its extract EGCG has been reported from time to time [137]. An in vitro study shows that high doses of EGCG induce mitochondrial outer membrane damage and uncoupling of oxidative phosphorylation in rat hepatocytes [138].

Chemical modification of EGCG, reliable delivery system and combination with other drugs seem to be feasible strategies to improve the therapeutic potential of EGCG. Adding a long acyl chain(C16-18) to EGCG was shown to increase its anti-influenza virus activity by 44-fold, and the chemical stability of EGCG was also enhanced by acylation. These acylated derivatives exhibit several times higher antibacterial activity than EGCG, especially against gram-positive organisms, and their antifungal MICs were also 2 to 4 times lower than EGCG [139]. Nanovesicle in situ gel based on EGCG phospholipid complex can improve the stability and utilization of EGCG and enhance the efficacy of caries prevention [140]. Adding 0.1%(w/w) EGCG to glass ionomer cement(GIC) can improve the mechanical and antibacterial properties of GIC without affecting its fluorine release property [141].

Although numerous phytochemicals have shown excellent antimicrobial activity in vitro, the activity of these drugs should be fully investigated in multi-bacterial models, especially for some bacteria that play an important role in co-infections, given the great resistance brought about by biofilm formation and the complex interactions among microbiota, such as the aforementioned *F. nucleatum* that can provide iron sources for itself and other periodontal disease-related microorganisms. On the other hand, considering the open nature of the oral environment and the complexity of host immunity, some significant differences observed in vitro may not necessarily cause appreciable changes in vivo. Whether EGCG can exert meaningful biological efficacy in vivo must be rigorously and long-term evaluated using in vivo experiments. There are still major deficiencies in these two aspects, which should be the focus of future research. In addition, it is also worthy of attention to explore the anti-infective effects of EGCG combined with other chemical drugs or oral materials, develop targeted or controlled release systems of EGCG and its derivatives, develop better in vivo models and standardize of technical procedures in vitro. More work is needed to apply EGCG as a routine anti-infective drug in clinical practice.
